# Cardiovascular protection of magnolol: cell-type specificity and dose-related effects

**DOI:** 10.1186/1423-0127-19-70

**Published:** 2012-07-31

**Authors:** Jennifer Hui-Chun Ho, Chuang-Ye Hong

**Affiliations:** 1 Graduate Institute of Clinical Medicine, Taipei Medical University, Taipei, Taiwan; 2 Center for Stem Cell Research, Wan Fang Hospital, Taipei Medical University, Taipei, Taiwan; 3 Institute of Engineering in Medicine, University of California, San Diego, La Jolla, CA, USA; 4 Department of Medicine, Wan Fang Hospital, Taipei Medical University, Taipei, Taiwan; 5 Department of Medicine, Wan Fang Hospital, Taipei Medical University, 111, Sec 3, Hsing-Long Rd, Taipei, 116, Taiwan

**Keywords:** Magnolol, Cardiomyocytes, Vascular endothelial cells, Smooth muscle cells, Inflammation, Antioxidant

## Abstract

*Magnolia officinalis* has been widely used in traditional Chinese medicine. Magnolol, an active component isolated from *Magnolia officinalis*, is known to be a cardiovascular protector since 1994. The multiplex mechanisms of magnolol on cardiovascular protection depends on cell types and dosages, and will be reviewed and discussed in this article. Magnolol under low and moderate dosage possesses the ability to protect heart from ischemic/reperfusion injury, reduces atherosclerotic change, protects endothelial cell against apoptosis and inhibits neutrophil-endothelial adhesion. The moderate to high concentration of magnolol mainly acts on smooth muscle cells and platelets. Magnolol induces apoptosis in vascular smooth muscle cells at moderate concentration and inhibits proliferation at moderate and high concentration. High concentration of magnolol also abrogates platelet activation, aggregation and thrombus formation. Magnolol also serves as an smooth muscle relaxant only upon the high concentration. Oral intake of magnolol to reach the therapeutic level for cardiovascular protection is applicable, thus makes magnolol an agent of great potential for preventing cardiovascular diseases in high-risk patients.

## Review

**Figure 1:**
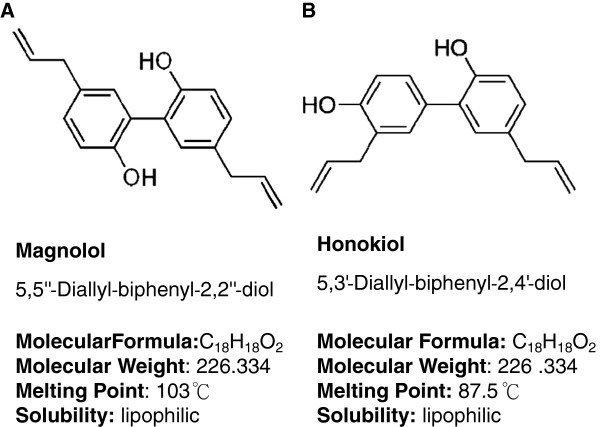
**Structure and chemical characteristics of magnolol and honokiol.** Magnolol (**A**) and honokiol (**B**) are isomers extracted from *Magnolia officinalis.* Both of magnolol and honokiol are lipophilic, biphenoid structure with molecular weight of 226.334. The melting point of magnolol is higher than that of honokiol.

Magnolol is an active component isolated from *Magnolia officinalis* (*Magnolia* bark). *Magnolia officinalis* is a traditional Chinese medicine widely used in facilitating bowel movement and ameliorate abdominal fullness. The bark is stripped from the stems, branches, and roots of Magnolia tree, and which is highly aromatic and polyphenolic components containing magnolol and honokiol (Figure 1) [1,2]. In addition to purification of magnolol from Magnolia-bark, preparation of synthetic magnolol/honokiol, its analogues and derivatives has been well established [3-6]. In the past decades, magnolol has been characterized as an anti-oxidant , anti-depressant, anti-allergic, anti-cancer and anti-microbial agent [7-11].

Using isolated rat heart mitochondria as an ex vivo model, Hong et al. demonstrated that magnolol exhibited free radical scavenging activities shown by the diphenyl-p-picrylhydrazyl assay, which was less potent than alpha-tocopherol (vitamin E) [12]. However, the ability of inhibiting ADP- or ferrous sulfate-induced heart mitochondrial lipid peroxidation from magnolol was 1000 times higher than which from alpha-tocopherol [12]. The lipid peroxidation inhibition ability by magnolol was not only found in isolated heart mitochondria, but also shown in preventing or treating rat from cecal ligation-induced sepsis by a dose-dependent manner from 10^-6^ to 10^-2^ mg/kg of magnolol via intravenous injection [13].

The potent antioxidant activities of magnolol and honokiol are thought to be the contribution of hydroxyl and allylic groups on a biphenolic moiety. The hydroxyl group on biphenolic moiety results in magnolol/honokiol against reactive oxygen species, inhibiting cell proliferation and antimicrobial activity [3,6,14]. It has been reported that most of allylated biphenolic magnolol/honokiol analogues possessed anti-proliferative activity and anti-MRSA capacity while magnolol analogues with flexible allylated biphenolic structure showed a better anti-virus activity than simple allylated ones [4,5]. In addition, the derivatives of honokiol with the biaryl structure bearing a hydroxyl and a allyl groups at the 4'-hydroxyl shown to be essential for neurite outgrowth-promoting activity [15].

The multiplex functional regulation by magnolol is a cell type specific effect. In this article, we will focus on tissues/cells involved in cardiovascular diseases, i.e. cardiomyocytes, endothelial cells, neutrophils, macrophages, platelets and smooth muscle cells in coronary artery and aorta. Literatures of magnolol research on cardiovascular protection, including our efforts, in the past 20 years will be reviewed and summarized in this article.

### Effects and molecular mechanisms of magnolol on cardiovascular system

The cardiovascular protection potentiality of magnolol through its antioxidant activity is first demonstrated by Hong et al. in 1994 [12]. It is well known that free radicals attack lipid membrane, protein and DNA. Excessive free radicals induce lipid peroxidation, protein denature and DNA damage, and that triggers cell death. In addition, vascular stenosis, cell death and inflammation are the major progressive factors to worse the cardiac function as well as vascular complications during cardiovascular dysfunction. In the past 20 years, magnolol has been found to have diverse functions in different cells of cardiovascular system. Those effects are dose-related, and are the consequence of different molecular mechanisms regulated by magnolol.

### Magnolol protects heart from myocardial infarction and ischemia/reperfusion injury

#### Magnolol reduces ventricular arrhythmia

In a series of animal studies, Hong and his team members demonstrated that intravenous injection of magnolol at the dosage above 10^-6^ mg/kg before coronary artery ligation successfully inhibited both ischemia- and reperfusion-induced ventricular tachycardia and ventricular fibrillation, while 10^-5^ mg/kg of magnolol and above significantly reduced the infarct size [16]. Honokiol had been found to more efficient for reducing ligation-induced infarct size (>10^-6^ mg/kg) but less sensitive to ventricular arrhythmia inhibition (at the dosage of 10^-4^ mg/kg) than magnolol [17]. Furthermore, to explore the mechanism of ventricular arrhythmia inhibition by magnolol, pretreatment of nitric oxide inhibitor (L-NAME) or cyclooxygenase inhibitor (aspirin) before ligation demonstrated that nitric oxide synthesis may also be involved in the anti-arrhythmic effect of magnolol or honokiol (10^-7^ mg/kg) [18].

#### Magnolol inhibits neutrophil infiltration in myocardium and restores systolic wall thickening fraction

Magnolol protected rabbit myocardium against coronary artery ligation-induced stunning evidenced by significantly enhancing the recovery of systolic wall thickening fraction 60 minutes after coronary artery reperfusion at the dosage from 10^-4^ to 10^-3^ mg/kg, relatively higher than which of reducing ventricular arrhythmia [19]. Under such concentration (2–5 x 10^-4^ mg/kg), magnolol could also prevent rat myocardium from ischemia/reperfusion injury and neutrophil infiltration [20].

#### Magnolol promotes coronary vasodilatation and inhibits myocardium apoptosis

Magnolol has been reported to possess the ability of reducing coronary arterial resistance after ligation under a high dosage (10^-1^ mg/kg) by measuring the rabbit coronary vascular resistance using pulsed Doppler velocimetry [21], suggesting that the underlying mechanism of magnolol responsible for coronary vasodilatation and myocardium protection is independent. Except for coronary vasodilatation, high concentration of magnolol at 10 mg/kg via intraperitoneal injection also demonstrated the ability to prevent myocardial ischemia and reperfusion injury associated cardiomyocytes apoptosis through enhancing the activation of ERK1/2 and modulation of the Bcl-xl proteins [22]. However, the regulation of ERK1/2 signaling pathway by magnolol shows a reciprocal effect in cardiomyocyte and cardiofibroblast. For rat cardiac fibroblast, on the contrary, magnolol (10 μM) significantly inhibited urotensin-II-induced proliferation through inhibiting ERK1/2 activation and interfering with ROS generation [23]. The *in vitro* results from cardiac fibroblasts indicate that magnolol may have a potential to decrease cardiac fibrotic change during the regenerative stage after infarction.

### Magnolol prevents atherosclerosis and vessel restenosis

#### Magnolol attenuates postangioplastic restenosis

Hyperlipidemia, resulting in atherosclerotic change, is one of the major risk factors for myocardial infarction and postangioplastic restenosis. In 2001, using balloon injury in a hyperlipidemic rabbit, Chen et al. demonstrated that daily intramuscular injection of 10^-3^ mg/kg magnolol for 6 weeks attenuated intimal thickening and MCP-1 expression induced by balloon denudation. Such effect of magnolol was related to inhibition of LDL oxidation rather than decreasing plasma lipid level [24], showing that lipid peroxidation inhibition by magnolol plays a role to prevent vessel restenosis.

#### Magnolol induces apoptosis and inhibits proliferation in vascular smooth muscle cells

Although ox-LDL contributes to vessel stenosis, intimal thickening is a direct consequence of vascular smooth cells proliferation. In experimental studies, magnolol was found to inhibit intimal hyperplasia induced by high-cholesterol diet [24]. Next, several research groups work on investigating the direct effect of magnolol on vascular smooth muscle cells instead of LDL peroxidation.

Magnolol (5 to 20 μM) *per se* dose-dependently induced apoptosis in vascular smooth muscle cells by increasing DNA fragmentation and activating caspase-3 and −9 activities. Magnolol reduced mitochondrial membrane potential which was Bcl-2 dependent [25]. However, under serum or TNFα stimulation, vascular smooth muscle cell proliferation can be abrogated by magnolol. Wu et al. reported that magnolol at the concentration of 50 μg/ml (200 μM) significantly reduced vascular smooth muscle cells progressing to the S-phase when DNA synthesis was triggered by serum. The proliferation inhibition by magnolol was also associated with reducing malondialdehyde formation, downregulation of NF-κB and increasing caspase-3 [26], suggesting that the proliferation inhibition may a net result from G1 cell cycle arrest, anti-oxidation and apoptosis induction by magnolol. Indeed, in the following research by Kim's group, 5 to 20 μM of magnolol concentration-dependently inhibited TNFα-induced vascular smooth cell proliferation through reducing ERK1/2 activity. Inhibition of ERK1/2 activity led to G1 cell cycle arrest which was a consequence of p21 upregulation and subsequent decrease in CDK-2 and CDK-4. Hence, magnolol also decreased MMP-9 promoter activity in vascular smooth muscle cells in response to TNFα, and which was transcriptional regulated by NF-κB and AP-1 [27].

### Magnolol promotes vessel dilation

In addition to affect on smooth muscle cell numbers, magnolol also works as a smooth muscle relaxant. In 1975, magnolol was found to act as a centrally acting muscle relaxant [28], such an effect was referred to magnolol with two hydroxyls into 2- and 2'-position of diphenyl molecule [29]. In 1990, Teng et al. found that high concentration as 10–100 μg/ml (40–400 μM) of magnolol efficiently blocked norepinephrine- or high K^+^-induced contraction of rat aorta in the present of endothelium [30]. Since that time, endothelium-derived relaxing factor and voltage-gated Ca^2+^ channels are thought to be the targets of magnolol in releasing smooth muscle tone. Further studies reported that both magnolol and honokiol at the concentration from 0.1 to 100 μM irreversibly abrogated carbachol- and high K^+^-induced smooth muscle contractions in porcine trachea and rat uterine without affecting the basal muscle tension. The mechanism of smooth muscle tone inhibition by magnolol directed to the blockade of Ca^2+^ influx through voltage-operated Ca^2+^ channels instead of Ca^2+^ release from intracellular Ca^2+^ stores [31,32].

### Magnolol inhibits vascular endothelial cell death

Magnolol shows reciprocal regulation on ERK1/2 activity in cardiomyocyte and fibroblast. Alternatively, magnolol inhibits the activation of NF-κB signaling pathway resulting in different effects on vascular smooth muscle cells, endothelial cells and neutrophils.

For vascular smooth muscle cells, magnolol induces cell death via activation of intrinsic (mitochondrial) apoptosis; however, for vascular endothelial cells, magnolol protects the cell viability through inhibition of intrinsic apoptosis. Using copper-induced ox-LDL to trigger intrinsic apoptosis, magnolol (2.5 to 20 μM) showed the protective effect on vascular endothelial cells via removal of intracellular ROS, so that ROS-induced cytochrome *c* releasing, caspase-3 activation as well as NF-κB activation was inhibited [33]. Honokiol, similar to magnolol, protect vascular endothelial cells against apoptosis. In an *in vitro* model of hyperglycemia-induced endothelial damage, honokiol (0.125 to 1 μM) suppressed NF-κB regulated COX-2 upregulation and intrinsic apoptosis [34]. Although it has been demonstrated that magnolol protects endothelial cells from the ox-LDL-induced apoptosis, the endothelial protection with magnolol *per se* is still unclear so far.

### Magnolol inhibits acute inflammation

#### Magnolol inhibits macrophage activation and suppresses neutrophil aggregation, activation and migration

In LPS-activated macrophage, both magnolol and honkiol inhibited iNOS expression and TNFα releasing. However, honokiol (IC50 6.4 μM) showed stronger inhibition effect on macrophage than magnolol (IC50 16.8 μM) [35].

During acute cardiovascular injury, neutrophil aggregation, activation, migration, endothelial adhesion and infiltration initiate a series of inflammatory responses which worse the cardiovascular function. Magnolol (30–90 μM) *per se* induced cytosolic-free Ca^2+^ elevation in neutrophil by stimulating Ca^2+^ release from internal stores and Ca^2+^ influx across the plasma membrane via activating inositol trisphosphate signalling pathway [36]. Upon PMA stimulation, magnolol at low concentration (0.1-10 μM) dose-dependently diminished PMA-induced Mac-1 (CD11b/CD18) upregulation in neutrophil and reduced sequential adhesion ability. Such effect was similar to exogenous superoxide dismutase or catalase showing that magnolol served as an anti-oxidant in abrogating neutrophil adhesion [37]. Furthermore, *in vivo* study demonstrated that magnolol suppressed cytosolic PKC activity in [3 H]phorbol 12,13-dibutyrate-labeled neutrophil rather than rat brain PKC activity with an IC 50 of 24.2 ± 1.7 μM. The attenuation of PKC activity in neutrophil by magnolol was responsible for its inhibition of neutrophil aggregation [38]. Hence, magnolol at 5–50 μM showed significantly suppress fMLP-activated human neutrophil migration in a concentration-dependent manner [20]. The above findings support that magnolol directly regulates neutrophil in an inflammatory environment.

#### Magnolol reduces neutrophil-endothelial adhesion

The next issue should be answered is whether magnolol can block the neutrophil-endothelial adhesion. For vascular endothelial cells, suppression of NF-κB regulated signals by magnolol leaded to anti-apoptotic effect [33,34]. It is also known that activation of NF-κB signaling pathway in vascular endothelial cells also results in both pro-inflammatory cytokines/chemokines releasing and cell adhesion molecules expression such as VACM-1, ICAM-1 and endothelial cell selectin [39], that promotes neutrophil-endothelial adhesion. In 2002, Chen et al. demonstrated that magnolol (5 and 10 μM) selectively inhibited TNFα-induced VACM-1 expression by abrogating nuclear translocation of NF-kB p65 in human aortic endothelial cells. They further confirmed that *in vivo* magnolol (10^-3^ mg/kg daily via intramuscular injection) attenuated the intimal thickening, TNFα and VCAM-1 protein expression in the thoracic aortas of cholesterol-fed rabbits [40]. Besides, magnolol at the concentration above 20 μM completely abolished IL-6-induced STAT3 phosphorylation in bovine aortic endothelial cells, and further downregulated ICAM-1, cyclin D1 and MCP-1 expression. The downregulation of ICAM-1 by magnolol resulted in suppression of IL-6-induced monocytic cells adhesion to endothelial cells [41].

### Magnolol prevents platelet aggregation and thrombus formation

The anti-platelet effect of magnolol and honokiol has been known for more than 20 years. Teng et al. firstly reported that magnolol and honokiol inhibited thromboxan B2 formation as well as intracellular calcium mobilization in platelets caused by collagen, arachidonic acid or thrombin [42]. Besides, collagen-induced platelet serotonin release was also inhibited by magnolol (3–30 μM) [43]. Using TCA method to measure the radioactive product and albumin in rat spleen microsomes or membrane fractions of human PMNs, magnolol and honokiol significantly inhibited the activity of acetyl-CoA: 1-alkyl-sn-glycero-3-phosphocholine acetyltransferase, a key enzyme in the biosynthesis of platelet-activating factor (IC50 60–150 μM) [44].

### Pharmacodynamics, Pharmacokinetics and Safety Test of Magnolol

The pharmacokinetics of magnolol are linear *in vivo* because that there were no significant difference in elimination half-life and total body clearance between intravenous injection and intravenous infusion [45]. The linear pharmacokinetics was found from a dose of 2 to 10 mg/kg via intravenous injection [46]. After rectal administration of magnolol (24.4 mg/kg) and honokiol (13.5 mg/kg), respectively, the linear concentration-time profiles of magnolol (from 40 to 400 ng/ml) and honokiol (from 20 to 200 ng/ml) in rat plasma could also be detected using a HPLC method [47]. However, LC/MS offered a larger range of linear concentration-time profiles of both magnolol and honokiol from 3.13 to 800 ng/ml [48].

In experimental studies, intravenous injection of magnolol at the dosage of 2–10 mg/kg revealed the C*max* around 10 μg/ml (40 μM), while the C*max* of 20 mg/kg magnolol via oral administration was 0.1 μg/ml (0.4 μM). The oral bioavailability of magnolol was around 4–5% [49,50]. The elimination half-life of magnolol was around 15 minutes and total body clearance was 72–75 ml/min/kg [50,51]. Tissue distribution of magnolol was predominantly in the liver, kidney, brain, lung, and heart after oral administration [50]. In healthy human subject, oral administration of magnolol could retain in plasma for more than 1 hour [51].

The safety test of magnolol or extracts of *Magnolia* bark has been reported. In pre-clinical study, oral administration (mice: 0.625-2.5 g/kg; rat: 0.06-0.48 g/kg/day for 21 days or 0.06-0.24 g/kg/day for 90 days) of ethanol extracts (94% magnolol and 1.5% honokiol) of *Magnolia* bark neither induced drug-related side effects nor altered immune response [52,53]. However, oral administration of 5–10 g/kg of *Magnolia* bark extracts for 14 days decreased liver and renal function in rat [54]. Daily oral intake 7.5 g of Hangekobokuto (containing 3 g *Magnolia* cortex per 17 g of Hangekobokuto) before meal for 2 weeks did not change the gastrointestinal symptom rating scale for healthy group and improved gastrointestinal symptoms in patients with functional dyspepsia [55]. A randomized, double-blind, placebo-controlled clinical study showed that oral administration of capsuled extracts of *Magnolia officinalis* and *Phellodendron amurense* (250 mg, three times a day for 6 weeks) was well tolerated in both healthy and obese patients, and regulation of cortisol only in obese patients was a benefit for weight control [56].

### Summary

**Figure 2:**
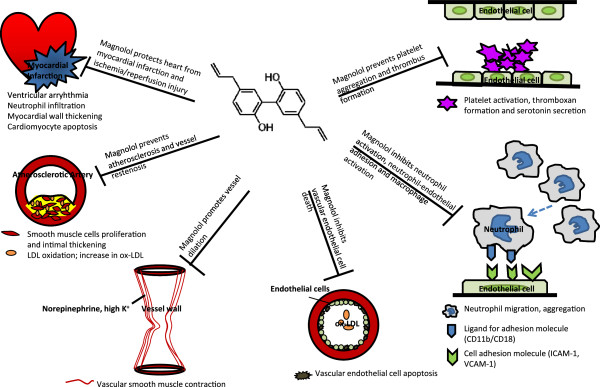
**Schema of magnolol in cardiovascular protection.** Magnolol protects heart from ischemic/reperfusion heart injury, reduces atherosclerotic change and endothelial cell apoptosis, inhibits neutrophil activation/adhesion and vascular smooth muscle cell proliferation, prevents platelet aggregation and thrombosis, and promotes vessel relaxation under different dosages.

Neutrophil infiltration, change of microRNA regulation, and activation of innate immunity and subsequent pro-inflammatory cytokine releasing as well as increasing oxidative stress in response to myocardial ischemic/reperfusion injury lead to cardiac arrhythmia and myocardial contractile dysfunction [57-59]. Endothelial damage triggers inflammation reaction and atherosclerotic change, platelet aggregation and thrombus formation, while mediators released by inflammatory cells promote vascular smooth muscle cell proliferation, all of which result in vascular occlusion [60-62]. The cardiovascular protections of magnolol result from attenuating ischemic/reperfusion heart injury, reducing atherosclerotic change and endothelial cell apoptosis, inhibiting neutrophil activation/adhesion and vascular smooth muscle cell proliferation, preventing platelet aggregation and thrombosis, and promoting vessel relaxation (Figure 2), and such cardiovascular protection effects regulated by magnolol are cell type specific and dose-related. In this article, we define that magnolol concentration at 10^-7^-10^-4^ mg/kg *in vivo* or ≦ 1 μM *in vitro* acts as a low dosage, 10^-4^-10^-2^ mg/kg *in vivo* or 1–100 μM *in vitro* is the moderate dosage, while ≧10^-1^ mg/kg *in vivo* or ≧100 μM *in vitro* will be the high dosage (Tab 1 and 2).

In general, lipid peroxidation inhibition ability of magnolol is linear, dose-dependent in physiologically low and moderate dosages. Under low dosage, anti-oxidation capacity of magnolol results in preventing myocardial infarction-induced ventricular arrhythmia and reducing infarct area. Under moderate dosage, the lipid peroxidation inhibition by magnolol protects myocardium from ischemic/reperfusion injury and neutrophil infiltration, attenuates postangioplastic restenosis and atherosclerotic change. Moderate concentration of magnolol also reduces vascular endothelial cell apoptosis via inhibiting NF-κB signaling pathway. Besides, the anti-inflammation ability of magnolol is promising under moderate concentration resulting in abrogating neutrophil Mac-1 mediated adhesion ligand expression, PKC-associated migration, macrophage activation and neutrophil-endothelial adhesion by reduction of VACM-1 and ICAM-1 expression via inhibiting NF-κB and STAT3 signaling pathway, respectively.

Moderate to high concentration of magnolol acts on vascular smooth muscle cells. Moderate concentration of magnolol triggers intrinsic apoptosis while moderate and high dosage further inhibit vascular smooth muscle cell proliferation through decreasing ERK1/2 activity. In addition, inhibition of platelet activation, aggregation as well as thrombus formation by magnolol are observed under the moderate to high concentration. It is worth to notify that only a high dosage of magnolol can release smooth muscle tone and subsequently lead to vasodilatation via regulation of Ca^2+^ channel. All the cell type specific and dose-related effects of magnolol on cardiovascular protection have been summarized in Tables 1 and 2.

**Table 1:** **Summary of *in vivo *magnolol effect on cardiovascular system**

**Dosage**	**Effect**	**Reference**
**Low (10**^**-7~**^**10**^**-4**^** mg/kg)**		
10^-7~−6^ mg/kg (i.v.)	Prevent post MI‒induced ventricular arrythmia	[16,18]
10^-5^ mg/kg (i.v.)	Decrease MI‒induced infarct size	[16]
**Moderate (10**^**-4~**^**10**^**-2**^** mg/kg)**		
10^-4~−3^ mg/kg (i.v.)	Protect myocardium against MI‒induced myocardial wall thinning	[19]
2-5 × 10^-4^ mg/kg (i.v.)	Decrease neutrophil infiltration in infarct heart	[20]
10^-3^ mg/kg (i.m.) daily	Attenuate balloon‒induced intimal thickening and inhibit LDL oxidation	[24]
10^-3^ mg/kg (i.m.) daily	Attenuate hypercholesteremia‒induced intimal thickening, TNFα and VCAM‒1 elevation in an aorta.	[40]
**High (≧10**^**-1**^** mg/kg)**		
10^-1^ mg/kg (i.v.)	Reduce coronary artery resistance after MI	[21]
10 mg/kg (i.p.)	Prevent MI‒induced cardiomyocyte apoptosis	[22]

i.v.: intravenous injection; i.m.: intramuscular injection; i.p.: intraperitoneal injection.

**Table 2:** **Summary of *in vitro *magnolol effect on cardiovascular system**

**Concentration**	**Effect**	**Reference**
**Low (**≦**1 μM) to Moderate**		
0.1‒10 μM	Diminish PMA‒induced neutrophil activation and reduce neutrophil adhesion ability	[44]
**Moderate (1–100 μM)**		
5‒10 μM	Inhibited TNFα‒induced VACM‒1 expression in aortic endothelial cells	[37]
10 μM	Inhibit proliferation of cardiac fibroblasts	[23]
16.8 μM	Inhibit LPS‒induced macrophage activation	[35]
5‒20 μM	Induce intrinsic apoptosis in vascular smooth muscle cells	[25]
5‒20 μM	Inhibit TNFα‒induced vascular smooth muscle cell proliferation	[27]
>20 μM	Downregulate IL‒6‒induced ICAM‒1 expression in endothelial cells and suppress monocyte adhesion to endothelial cells	[41]
2.5‒20 μM	Inhibit copper‒induced ox‒LDL triggered endothelial cell apoptosis	[33,34]
24.2 μM	Inhibit neutrophil aggregation	[38]
3‒30 μM	Inhibit collagen‒induced platelet serotonin release	[43]
5‒50 μM	Suppress fMLP‒activated neutrophil migration	[20]
30‒90 μM	induce cytosolic‒free Ca2+ elevation in neutrophil	[36]
**Moderate to High (**≧**100 μM)**		
200 μM	Reduce serum‒induced vascular smooth muscle cell proliferation	[26]
40‒400 μM	block norepinephrine‒ or high K^+−^induced contraction of aorta	[30]
60‒150 μM	Inhibit biosynthesis of platelet‒activating factor from PMNs	[44]

## Conclusion

Multifunction of magnolol on cardiovascular system suggests the great potential of such a traditional Chinese medicine in treating or preventing cardiovascular diseases. From the clinical point of view, all the cardiovascular protection *in vivo* by magnolol can be observed under or below the dosage of 0.1 mg/kg via intravenous injection according to those above research achievements. To reach the therapeutic level through oral administration with 5% of oral bioavailability, 2 mg/kg per day, i.e. daily 120 mg of magnolol for a 60-kg adult, may be sufficient for cardiovascular protection, and such a dosage is applicable. We conclude that magnolol is a non-steroid and non-aspirin compound with strong cardiovascular protection ability, which has a great potential of preventing cardiovascular insults under daily intake in high-risk patients. Further clinical trials for investigation of safety and therapeutic range of magnolol in a healthy and a high-risk group should be warranted.

## Abbreviations

MRSA, Methicillin Resistant Staphylococcus Aureus; GABA, γ-aminobutyric acid; LTB4, Leukotriene B4; LTC4, Leukotriene C4; IgE, Immunoglobulin E; S. aureus, Staphylococcus aureus; DNA, Deoxyribonucleic acid; ADP, Adenosinediphosphate; L-NAME, L-N G-nitro-arginine methyl ester; ERK1/2, Extracellullar signal-regulated kinase 1/2; Bcl-xl, B-cell lymphoma-extra large; ROS, Reactive oxygen species; MCP-1, Monocyte chemotactic protein-1; LDL, Low density lipoprotein; ox-LDL, Oxidized low density lipoprotein; Bcl-2, B-cell lymphoma 2; TNFα, Tumor necrosis factor-alpha; NF-κB, Nuclear factor kappa-light-chain-enhancer of activated B cells; CDK, Cyclin-dependent kinase; MMP-9, Matrix metalloproteinase-9; AP-1, Activation protein-1; K, Potassium; Ca, Calcium; COX-2, Cyclooxygenase-2; PMA, Phorbol-12-myristate-13-acetate; PKC, Protein kinase C; f-MLP, N-formylmethionyl-leucyl-phenylalanine; VACM-1, Vascular cell adhesion molecule-1; ICAM-1, Intercellular cell adhesion molecule-1; IL-6, interleukin-6; LPS, Lipopolysaccharide; iNOS, Inducible nitric oxide synthetase; STAT3, Signal transducer and activator of transcription protein 3; TCA, Trichloroacetic acid; PMNs, Polymorphonuclear leukocytes; HPLC, High-performance liquid chromatographic; LC/MS, Liquid chromatography tandem mass spectrometry; Cmax, Maximal plasma concentration.

## Competing interests

The authors declare that they have no competing interests.

## Authors’ contributions

JH carried out the design, acquisition, analysis and interpretation of data, drafting the manuscript. CY had contributed to conception, design and critical version of important intellectual content and final approval of the manuscript. All authors read and approved the final manuscript.

## Authors’ information

Dr. Jennifer Hui-Chun Ho is the Director of Center for Stem Cell Research and the Deputy Director of Medical Research and Education at Wan Fang Hospital, Taipei Medical University, and is also an Associate Professor at Graduate Institute of Clinical Medicine, Taipei Medical University. The main research work in her lab is using small molecule or physical stimulus to modify stem cell activity and enhance the efficacy of stem cell transplantation.

Dr. Chuang-Ye Hong was the formal Superintendent of Wan Fang Hospital, Taipei Medical University and also the Professor at Graduate Institute of Clinical Medicine, Taipei Medical University and a consultant cardiologist at Wan Fang Hospital. Prof. Hong is an expert in pharmacological research of medical herbs on cardiovascular diseases. Prof. Hong was the Director of Institute of Traditional Medicine at National Yang-Ming University from 1992 to 1997, when he led a research team working on translational research of traditional Chinese medicine, especially magnolol and honokiol.
